# Safety of Bacillus Calmette-Guérin Vaccination and Impact on HIV-1 Latent Reservoir Size in People With Treated HIV-1 Infection

**DOI:** 10.1093/ofid/ofaf611

**Published:** 2025-09-29

**Authors:** Emily West, Sandra E Chaudron, Doris Russenberger, Christina Grube, Karin J Metzner, Kathrin Neumann, Jasmin Tschumi, Marisa Kälin, Terence K Tutumlu, Cédric Dollé, Roger D Kouyos, Huldrych F Günthard, Dominique L Braun, Johannes Nemeth

**Affiliations:** Department of Infectious Diseases and Hospital Epidemiology, University Hospital Zurich, University of Zurich, Zurich, Switzerland; Department of Infectious Diseases and Hospital Epidemiology, University Hospital Zurich, University of Zurich, Zurich, Switzerland; Department of Infectious Diseases and Hospital Epidemiology, University Hospital Zurich, University of Zurich, Zurich, Switzerland; Department of Infectious Diseases and Hospital Epidemiology, University Hospital Zurich, University of Zurich, Zurich, Switzerland; Department of Infectious Diseases and Hospital Epidemiology, University Hospital Zurich, University of Zurich, Zurich, Switzerland; Institute of Medical Virology, University of Zurich, Zurich, Switzerland; Department of Infectious Diseases and Hospital Epidemiology, University Hospital Zurich, University of Zurich, Zurich, Switzerland; Department of Infectious Diseases and Hospital Epidemiology, University Hospital Zurich, University of Zurich, Zurich, Switzerland; Institute of Medical Virology, University of Zurich, Zurich, Switzerland; Department of Infectious Diseases and Hospital Epidemiology, University Hospital Zurich, University of Zurich, Zurich, Switzerland; Department of Infectious Diseases and Hospital Epidemiology, University Hospital Zurich, University of Zurich, Zurich, Switzerland; Department of Infectious Diseases and Hospital Epidemiology, University Hospital Zurich, University of Zurich, Zurich, Switzerland; Department of Infectious Diseases and Hospital Epidemiology, University Hospital Zurich, University of Zurich, Zurich, Switzerland; Institute of Medical Virology, University of Zurich, Zurich, Switzerland; Department of Infectious Diseases and Hospital Epidemiology, University Hospital Zurich, University of Zurich, Zurich, Switzerland; Institute of Medical Virology, University of Zurich, Zurich, Switzerland; Department of Infectious Diseases and Hospital Epidemiology, University Hospital Zurich, University of Zurich, Zurich, Switzerland; Institute of Medical Virology, University of Zurich, Zurich, Switzerland; Department of Infectious Diseases and Hospital Epidemiology, University Hospital Zurich, University of Zurich, Zurich, Switzerland

**Keywords:** BCG, HIV, latent reservoir, safety, tuberculosis

## Abstract

**Background:**

Bacillus Calmette-Guérin (BCG) vaccination, used against tuberculosis, is recognized for its immunomodulatory properties, a phenomenon referred to as “trained immunity.” Given these effects, there is increasing interest in evaluating its safety and impact on immune function in people living with HIV-1 (PWH). Historically, BCG was contraindicated in PWH due to safety concerns in immunocompromised individuals. This study aims to assess both the safety of BCG in PWH and its effects on the HIV-1 latent reservoir size.

**Methods:**

This Phase IIA randomized, double-blind, placebo-controlled, single-center trial enrolled 60 PWH with a suppressed viral load and CD4 T-cell count >350/μL. Participants were randomized in a stepped-wedge design into equal groups for early or late BCG vaccination. Each participant received a single intradermal dose of BCG vaccine followed by a placebo 3 months later, or vice versa. The HIV-1 latent reservoir was quantified at 3-month intervals to day 270. The primary endpoint was the HIV-1 reservoir size 6 months postvaccination, with secondary endpoints including safety outcomes.

**Results:**

No significant differences were found in intact proviral HIV-1 DNA levels at 6 months compared to baseline. Local reactions occurred in 96% of participants, leading to scarring in 73%. No systemic infections or serious BCG-related adverse events were observed.

**Conclusions:**

BCG vaccination is safe in PWH, but local skin reactions including scarring are common. There was no significant effect on the HIV-1 reservoir. These findings provide valuable insights into the safety profile of BCG vaccination in PWH, emphasizing its potential for broader immunological studies.

Bacillus Calmette-Guérin (BCG), a live attenuated vaccine developed over a century ago for the prevention of tuberculosis (TB), has demonstrated broader immunomodulatory effects beyond its primary use [1]. These effects, termed “trained immunity,” involve the long-term functional reprogramming of innate immune cells, enhancing their capacity to respond robustly to subsequent infections through epigenetic and metabolic alterations [2–7]. While BCG's potential to modulate immune responses has been widely studied in various infectious diseases, its safety and effects in people living with HIV-1 (PWH) remain unexplored [8, 9].

Historically, BCG vaccination has been contraindicated in PWH due to concerns about adverse outcomes, particularly the risk of *Mycobacterium bovis* dissemination in immunocompromised individuals [8]. Advances in antiretroviral therapy (ART) have enabled most PWH to achieve viral suppression and substantial immune restoration, warranting a re-evaluation of the vaccine's safety in this population [10] due to the epidemiological overlap between high HIV and TB prevalence. Previous studies on BCG and HIV have yielded conflicting results, likely due to substantial variation in study settings and populations [11–14]. Differences in BCG vaccine strain, host genetic background, ART status, comorbidities, demographic characteristics, and environmental exposure to mycobacteria may all influence outcomes. Our study is, to our knowledge, the first to systematically evaluate BCG vaccination in PWH on long-term ART in a low-endemic setting, providing a controlled framework to begin disentangling these complex interactions.

The HIV latent reservoir, comprising infected cells harboring replication-competent proviruses, persists despite ART, necessitating lifelong treatment to prevent viral rebound and disease progression [15, 16]. Interventions that enhance immune activation and clearance of these reservoirs are of significant interest [17, 18]. BCG's trained immunity effects have shown promise in modulating responses to viral infections, as evidenced by reduced viremia in vaccinated individuals during yellow fever outbreaks and other viral challenges [19, 20]. Our rationale for assessing the effect of BCG vaccination on the HIV proviral reservoir in individuals on suppressive ART was based on several indirect yet converging lines of evidence. Latent *Mycobacterium tuberculosis* (MTB) infection has been associated with lower viral set points in people with HIV [21], along with measurable alterations in innate immune activation and substantial remodeling of the adaptive immune response. While these observations were made in the absence of ART, they suggest that mycobacterial exposure may influence reservoir dynamics. Given the ethical and practical constraints, the setting of chronic, treated HIV infection is currently the only context in which such interventions can be safely explored. We therefore used BCG as a surrogate for latent MTB exposure and designed this study with two central aims. The first was to determine whether BCG vaccination has an impact on the size of the HIV latent reservoir, potentially contributing to novel strategies for reservoir reduction. The second was to evaluate the safety of BCG vaccination in PWH with suppressed HIV viral loads and CD4 T-cell counts indicative of immune restoration. By systematically investigating the safety and immunological effects of BCG in this context, this study aims to fill a critical gap in understanding and explore its potential role in HIV cure strategies.

## METHODS

### Study Population

We recruited patients at our center with stable suppressed viral load under antiretroviral treatment from a subcohort within the Swiss HIV Cohort Study [10], known as the Systems-X Project [22]. Systems-X had previously investigated 1057 patients with repeated measurements of total HIV-1 DNA levels during a median of 5.4 years on ART. These patients were found, on average, to have slowly declining total HIV-1 DNA levels, which tended to plateau after 5 years on ART. By selecting participants for the present study from among this cohort, we deliberately ensured a well-characterized group with, on average, a longitudinally stable total HIV-1 DNA.

In addition, patients needed to have a VL of <50 copies/mL at screening and to be older than 18 years. We excluded patients with a history of active TB, evidence of latent TB within the past 10 years, BCG vaccination within the past 10 years, a history of allergy to BCG, virological failure or ART interruption, with a CD4 count of <200 or between 200 and 350 with a CD4% <33%, who were immunosuppressed (e.g. on ≥20 mg prednisone daily or equivalent), had a current febrile illness at screening and women who were pregnant or breastfeeding.

### Study Design

**Figure 1.**
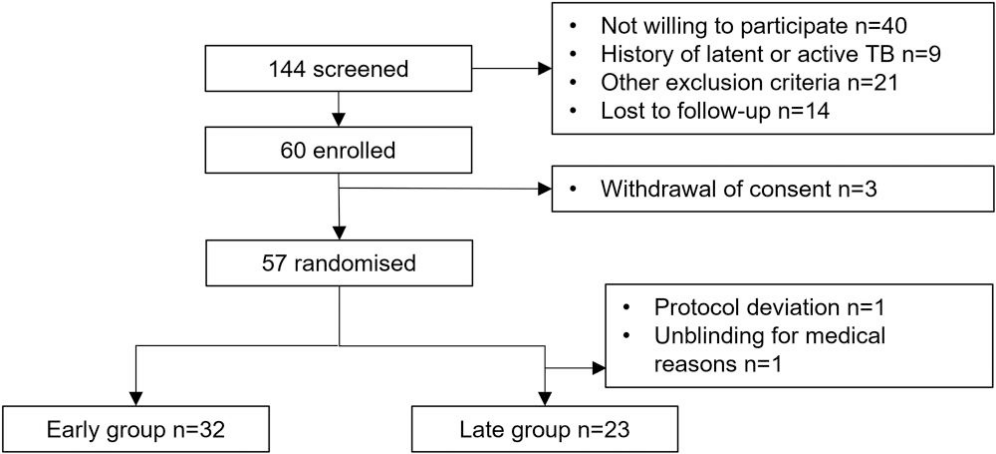
Trial overview.

For this double-blind placebo-controlled study, we employed a stepped-wedge design, randomizing participants into “early” and “late” administration groups and thus allowing each participant the possible benefits of receiving BCG (Figure 1). Participants were randomized to receive BCG Vaccine AJVaccines (*Mycobacterium bovis* Danish strain 1331) at a dose of 2–8 × 10^5^ colony-forming units, administered intradermally according to the manufacturer’s instructions, followed 3 months later by a 0.9% saline placebo (“early”), or vice versa (“late”). The stepped-wedge design facilitated comparison of the effects of BCG and placebo over 3 months postadministration in the early versus late groups and following the effect on reservoir size over a total of 9 months in the early group.

### Study Endpoints

The size of the HIV-1 reservoir was measured in each participant at baseline and then 3-monthly until day 270. We chose the primary endpoint of change in HIV-1 reservoir size within each individual from pre- to 6 months post-BCG administration. Our secondary endpoints were the effects of BCG and placebo on HIV-1 DNA (measured 3-monthly) over three months postadministration in the early versus late groups, and the effect over a total of 9 months in the early group. Patients were additionally monitored for viral load including blips and were asked during telephone interviews 2 and 14 days following each injection about predefined adverse events of interest including local and systemic reactions to BCG, which were graded by blinded investigators. Body temperature was measured in response to symptoms. Data on all serious adverse events were collected. Because BCG is already a licensed drug, we decided against collection of nonserious and laboratory adverse events.

### Quantification of the HIV-1 DNA

The HIV-1 proviral DNA was quantified using the intact proviral DNA assay (IPDA) on a digital PCR system [23]. DNA was isolated from a mean of 10.77 million (SD 1.11) peripheral blood mononuclear cells (PBMCs) using the AllPrep DNA/RNA Mini Kit (QIAGEN) on the QIAcube, following the manufacturer's protocol. Briefly, cells were pelleted by centrifugation at 300*g* for 5 min, the supernatant was removed, and the cell pellet was resuspended in Buffer RLT Plus. Lysates were homogenized using a QIAshredder spin column at 17,000*g* for 2 min and processed on the QIAcube using the “RNA_AllPrepDNARNAMini_AnimalTissuesAndCells_LargeSamplesPartAandB_V4” protocol, with DNA eluted in H_2_O. DNA was diluted 1:100 for CCR5 measurements, while undiluted DNA was reserved for HIV-1 quantification.

The IPDA was performed following the method described by Bruner et al on a Qiagen digital PCR system [24]. In brief, the IPDA targets two regions of the HIV-1 genome, shown to be conserved among intact proviruses, with two distinct fluorophores. Single positive reactions thus contain total proviruses (5′ total or 3′ total, respectively), while only double positive reactions contain intact proviruses. Since naturally and experimentally induced DNA fragmentation leads to artificially decreased detection of double positive reactions, a host gene needs to be quantified to assess DNA shearing. Here, CCR5 was quantified in an independent reaction with two amplicons spaced at the same distance as the HIV-1 amplicons. Double positive CCR5 reactions contain an unsheared gene fragment, while reactions positive for only one CCR5 fluorescence contain part of a sheared gene fragment. A DNA shearing index was calculated for every sample as the ratio of double positive CCR5 reactions over single positive reactions, and this index was applied to both CCR5 copy number input and HIV-1 positive reactions to correct for experimentally observed DNA shearing. Reactions were prepared with 1x Reaction Buffer (Qiagen), 0.8 μM forward and reverse primers (Microsynth, Balgach, Switzerland) (Table 1), 0.4 μM FAM-labeled TaqMan probes (Microsynth), and 10 μL of DNA. The cycling conditions were as follows: 95°C for 2 min, then 50 cycles of 95°C for 15 s and 59°C for 60 s.

**Table 1.** Primer Sequences for IPDA Assay

psi_IPDA_fw	CAGGACTCGGCTTGCTGAAG	psi
psi_IPDA_rv	GCACCCATCTCTCTCCTTCTAGC	psi
psi_IPDA_probe	FAM-TTTTGGCGTACTCACCAGT-3MGBEc	psi
env_IPDA_fw	AGTGGTGCAGAGAGAAAAAAGAGC	env
env_IPDA_rv	GTCTGGCCTGTACCGTCAGC	env
env_IPDA_intact_probe_wt	YY-CCTTGGGTTCTTGGGA-MGB-Q530	env
env_IPDA_hypermut	CCTTAGGTTCTTAGGAGC	env
mf51_655	TGCAGCTCTCATTTTCCATAC	CCR5
mf52	GAGTTTTTAGGATTCCCGAGTA	CCR5
mf73_YY	YY-CCGCTGCTGTCATGGTCATCTG-BHQ1	CCR5
CCR5_upstr_fw	GCCTAGACACCGATCTGAAAG	CCR5 upstream
CCR5_upstr_rv	GGCTGTCTACCTCATGGATTC	CCR5 upstream
CCR5_upstr_probe	FAM-CTCCCACCA/ZEN/GGTTTGAGCCTATCT-3IABkFQ	CCR5 upstream

The total HIV-1 reservoir size was calculated as the sum of shearing corrected 5′ total, 3′ total, and intact HIV-1 copy numbers per genomic equivalents.

### Statistical Analysis

Linear regression models were employed to adjust both the total and intact proviral DNA counts, accounting for the total number of genomic equivalents. The genomic equivalents were incorporated as natural splines to accommodate potential nonlinear relationships. To compare reservoir sizes across time points, paired *t*-tests were utilized for the total reservoir, given its normal distribution. For the intact reservoir, which did not follow a normal distribution, a Mann–Whitney *U* test was applied to assess differences.

### Patient Consent Statement

The local ethics committee (Ethikkommission Zürich) approved the clinical trial according to the 2008 Declaration of Helsinki principles with the identification number 2021–01481. Study participants provided written informed consent before enrolment. This study is registered with ClinicalTrials.gov, number NCT05004038.

## RESULTS

**Figure 2.**
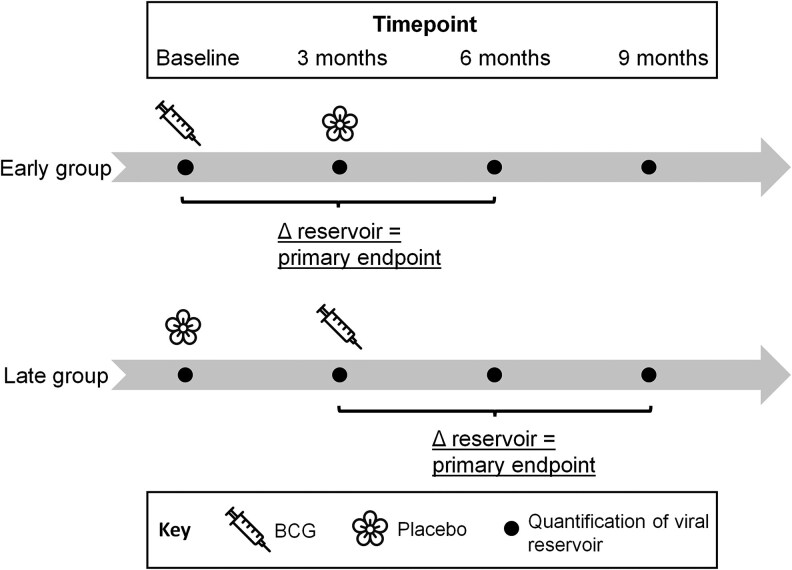
Trial profile.

From a total of 144 patients screened, 60 were recruited and randomized (Figure 2). Baseline characteristics of the study population are shown in Table 2. Participants were predominantly male (88%) with a median age of 54 years, had been on ART for an average of 15 years and had very high self-reported adherence to therapy, with 93% missing a tablet less frequently than once per month. The immunological status of participants was robust, with a median CD4 count of 620 (IQR 483.5–844) cells/µL.

**Table 2.** Baseline Characteristics

Category	Characteristic	Number or median (IQR)^a^
	Total	60
Sex	Male	53
	Female	7
Age	Age (y)	54 (47.75–60)
Risk	HET	15
	MSM	42
	IVDU	2
	Unknown	1
HIV-1 subtype	B	41
	Non-B	14
	Unknown	5
CD4 values	Baseline CD4 count (cells/µl)	620.5 (483.5–844)
	CD4 nadir (cells/µl)	194.5 (143–255.25)
HI viral load	Baseline VL (c/mL)	<20 (<20 - < 20)
	Zenith VL prior to ART start (c/mL)	83900 (33975–340000)
Prior opportunistic infections	CDC Group B and C	17
Coinfections	Syphilis	25
	Hepatitis C virus	5
	Cytomegalovirus	51
	Toxoplasmosis	23
Vaccination	BCG >10 y prior to enrolment	0
Years on ART	Years on ART prior to enrolment	15 (14–16.25)
ART regimen	INSTI-based	43
	NNRTI-based	8
	PI-based	9
	NRTI backbone	59
Missed ART doses	Never	56
	Once per month	3
	Once per 2 wks	1

^a^At or last recorded measurement prior to the time point of inclusion.

IQR, interquartile range; HET, heterosexual risk group; MSM, men who have sex with men risk group; IVDU, IV-drug use risk group; VL, viral load; CDC, centers for disease control and prevention; ART, antiretroviral therapy; INSTI, integrase strand transfer inhibitor; NNRTI, non-nucleoside reverse transcriptase inhibitor; NRTI, nucleoside reverse transcriptase inhibitor; PI, protease inhibitor.

There were 5 dropouts, 3 due to withdrawal of consent before randomization. One patient received a diagnosis of acute myeloid leukemia (AML) on day 56 following the second injection, and the decision was made to unblind this patient, revealing the second injection had been BCG. This prompted the search for *Mycobacterium bovis* in blood cultures, which, however, remained negative. Retrospectively, minor abnormalities of the full blood count associated with AML were already present at the time of BCG administration. At enrolment, the patient's blood count was normal with the exception of low monocytes and lymphocytes. By the time of the second injection, the patient had become neutropenic (neutrophil count 0.29), with the last value available before administration being 0.64. We regret to report this patient's death due to complications of AML 19 months following diagnosis and removal from the study.

Finally, one patient was included by mistake at a CD4 count of 169 cells/µl (19%). Due to this protocol deviation, he was immediately unblinded, revealing that he received placebo, and constituting the final drop-out. This patient had developed low CD4 levels in July 2021 due to treatment for prostate cancer, with randomization occurring in April 2022. Study procedures and follow-up were thus completed for a total of 55 patients.

### Adverse Events

Local reactions observed to BCG administration were almost universal, with 39% of BCG administrations resulting in only local erythema or induration, 34% in skin ulceration and 23% in necrosis or abscess formation, compared to 5, 2% and 0%, respectively for placebo (Table 3). There was scar formation after 73% of BCG administrations, none following placebo. None of the patients required antimycobacterial drugs or surgical drainage.

**Table 3.** Adverse Events

Adverse Event	Number Following BCG (%)	Number Following Placebo (%)
Injection site reaction (Grade)	…	…
None (0)	2 (3.6)	52 (92.8)
Erythema/induration (1)	22 (39.2)	3 (5.4)
Ulceration (2)	19 (33.9)	1 (1.8)
Abscess/necrosis (3)	13 (23.2)	0
Any reaction (1, 2, or 3)	54 (96)	4 (7)
Pain at injection site	9 (16.1)	0
Allergic reaction	0	0
Local lymphadenopathy	0	0
Fever >38.3 C	0	0
Headache	2 (3.6)	0
Osteitis/Osteomyelitis	0	0
Scar formation	41 (73.2)	0

This table includes information for 55 patients who completed the study and for one patient who dropped out on day 86 after the second injection.

There were few systemic symptoms following BCG, with only two patients reporting headache and none reporting fever. As detailed in Table 4, we recorded 12 serious adverse events during the study, none of which were deemed BCG-related and all of which resolved.

**Table 4.** Serious Adverse Events

Category of Serious Adverse Event	Description (Each Event Occurred Once)	Relatedness to BCG
Infectious	Perirectal abscess requiring drainage	Unrelated
	Neutropenic fever/sepsis	Unrelated
	COVID-19 infection in neutropenia	Unrelated
	COVID-19 with acute laryngopharyngitis	Unrelated
Malignant	New diagnosis of prostate adenocarcinoma	Unrelated
Traumatic	Fracture left humerus	Unrelated
	Boerhaave syndrome	Unrelated
	Anal pain due to foreign body	Unrelated
Cardiovascular	In-stent restenosis and thrombosis of right popliteal artery	Unrelated
	Acute right leg ischemia (in the same patient)	Unrelated
Other	ICD electrode dysfunction	Unrelated
	Alcohol intoxication	Unrelated

### Effect on HIV-1 Total and Intact Proviral HIV-1 DNA

**Figure 3.**
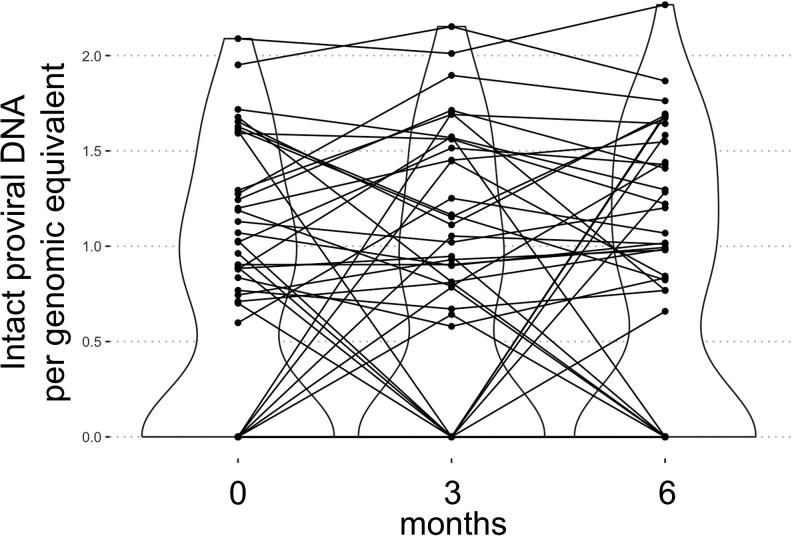
Intact HIV-1 latent reservoir. Intact proviral DNA in PBMC from *n* = 58 PWH over the study period. The intact proviral DNA assay (IPDA) was performed following the method described by Bruner et al. on a Qiagen digital PCR system [24]. Intact proviral DNA are expressed as counts per genomic equivalents. Time points are prior to BCG vaccination, 3 and 6 m after BCG vaccination.

A significant increase in total HIV-1 DNA was observed at 3 months post-BCG vaccination; however, this increase was no longer statistically significant at the primary endpoint 6-month mark. No significant changes were detected in the intact HIV-1 DNA over the same period (Figure 3). It should be noted however that a considerable fraction of intact HIV-1 DNA measurements (78/165) were below the detection limit, thereby reducing statistical power to detect differences. Five individuals demonstrated low-grade viremia, defined as measurable HIV-1 RNA levels between 20 and 50 copies/mL. Notably, the amount of intact HIV-1 DNA did not change in these individuals, consistent with observations in the broader cohort (Figure 3).

### Viral Load and Blips

Neither blips (defined as >50–200 copies/mL) nor viral failures (defined as >200 copies/mL) were significantly increased when comparing the trial period with the period 2 years prior to BCG vaccination. Similarly, CD4 and CD8 cell counts, CD4/CD8 ratio, total lymphocyte and total leukocyte counts remained unchanged when comparing the time periods prior to and following BCG vaccination.

## DISCUSSION

This study evaluated the safety and immune response outcomes of BCG vaccination in PWH with viral suppression and immune recovery. The results showed no cases of disseminated BCG infection, while localized skin reactions were a common occurrence. BCG remains the only approved vaccine for TB [11, 12, 25], and its safety profile in PWH with controlled HIV infection had not been systematically investigated in adults prior to this study. The size of the intact HIV reservoir remained stable throughout the study.

The findings of this study carry several implications. The demonstration of BCG's safety in PWH with well-controlled HIV infection provides valuable data, particularly given the historical concerns surrounding the use of live vaccines in this population [11]. While PWH remain at elevated risk for active TB, its application in adults, particularly in settings where BCG is not routinely recommended, requires careful consideration. The notable rate of significant local reactions, with abscess or necrosis following 23% of applications, highlights the need for caution. Any future exploration of BCG in PWH should carefully weigh the potential immunological insights against the local side effect profile and the lack of a clear clinical indication for its use in adults in most current settings [25]. The data provided here serve primarily for pathogenesis-related research rather than immediate clinical application [19, 26–29].

In our well-characterized patient group known to have a stable HIV reservoir on average, HIV RNA and intact HIV DNA levels remained stable, and we could not demonstrate a clear effect on the reservoir associated with HIV persistence. However, the observed increase in total HIV DNA at 3 months postvaccination, including a modest rise in intact HIV DNA, suggests that BCG vaccination may exert a prolonged activating effect on the latent HIV reservoir. While BCG is not a latency reversal agent in the classical sense, this sustained increase in intact HIV DNA could reflect a shift in reservoir dynamics—potentially enhancing the transcriptional activity or cellular turnover of latently infected cells. This observation raises the possibility that BCG might serve as a valuable adjunct to latency-reversing strategies in combination therapy or treatment interruption trials. The discrepancy between the observed increases in total and intact HIV DNA at 3 months might be explained by heightened immune activation preferentially eliminating productively rather than defectively infected cells. Given the transient nature of the signal and absence of adverse clinical outcomes, we do not interpret the increase as a safety concern but rather as a biological effect warranting further investigation.

Studies of the effects of other licensed vaccines on the HIV latent reservoir demonstrate relevant differences to our findings. Trivalent subunit influenza vaccination resulted in increased HIV RNA and decreased HIV DNA in a small cohort with treated HIV infection, suggesting activation of the HIV latent reservoir [30]. Studies involving SARS-CoV-2 mRNA vaccines, such as those developed by Pfizer-BioNTech and Moderna, have also demonstrated activation within the HIV latent reservoir, indicated by a transient release of HIV RNA in vitro [31]. This suggests that subunit and mRNA vaccines may stimulate latent HIV within immune cells. Our contrasting results may be explained by the different timing of sample collection within our study, which was deliberately chosen to be later in order to detect prolonged immune activation. In sum, BCG does not increase blips or the risk of viral failure from 6 to 12 weeks after administration in PWH, suggesting it does not compromise the efficacy of ART.

This study's strengths include its randomized controlled trial (RCT) design, double-blinding, and prospective approach with a long prevaccination observational period, which collectively enhance the robustness of the findings. The use of a comprehensively defined and homogenous population from the Systems-X Cohort with well-controlled HIV infection further strengthens the validity of the results.

The study also has limitations. The single-center design and relatively homogenous study population may limit the generalizability of the findings to broader, more diverse populations. Additionally, the study was conducted in a low-endemic country for TB [32], which may influence the applicability of the results to regions with higher TB prevalence. However, this homogeneity may also be considered a strength, as pathophysiological studies often benefit from examining more uniform populations, which can provide clearer insights into specific mechanisms. Additionally, the low transmission of TB and limited environmental exposure to nontuberculous mycobacteria in this setting likely resulted in a study population that was largely naive to antimycobacterial responses [32]. Consequently, the immune responses to mycobacteria observed in this cohort are likely to be relatively homogeneous, as they were less influenced by prior exposure, minimizing confounding effects and facilitating the interpretation of results. Potential biases in study conduct, such as selection bias or reporting bias, were minimized through rigorous trial design and execution, although they cannot be entirely excluded.

This study provides valuable evidence regarding the safety of BCG vaccination in people with well-controlled HIV infection, demonstrating no serious adverse events in this population. However, these findings should not be interpreted as a recommendation for the routine use of BCG in PWH. Rather, our findings support the use of BCG as a model to explore host responses to self-limiting mycobacterial exposure in PWH on ART. A multiomics investigation is ongoing to define the immunological pathways engaged by BCG in this setting. These data will help determine whether BCG might function as a latency-reversing agent or as an innate immune modulator—for example through NK cell activation—in future combination strategies aimed at HIV cure. Our findings serve as a foundation for further exploration rather than a basis for clinical application at this stage.

In conclusion, this study provides the first direct data on the effects of BCG in the setting of controlled HIV infection and suggests that immune activation induced by BCG may warrant further investigation, particularly as a component of combination strategies aimed at reducing the proviral reservoir.
